# Obesity risk is associated with brain glucose uptake and insulin resistance

**DOI:** 10.1530/EJE-22-0509

**Published:** 2022-10-26

**Authors:** Laura Pekkarinen, Tatu Kantonen, Eleni Rebelos, Aino Latva-Rasku, Prince Dadson, Tomi Karjalainen, Marco Bucci, Kari Kalliokoski, Kirsi Laitinen, Noora Houttu, Anna K Kirjavainen, Johan Rajander, Tapani Rönnemaa, Lauri Nummenmaa, Pirjo Nuutila

**Affiliations:** 1 Turku PET Centre, University of Turku, Turku, Finland; 2 Department of Endocrinology, Turku University Hospital, Turku, Finland; 3 Clinical Neurosciences, Turku University Hospital, Turku, Finland; 4 Turku PET Centre, Åbo Akademi University, Turku, Finland; 5 Institute of Biomedicine, Research Centre for Integrative Physiology and Pharmacology, University of Turku, Turku, Finland; 6 Department of Medicine, University of Turku, Turku, Finland; 7 Department of Psychology, University of Turku, Turku, Finland

## Abstract

**Objective:**

To investigate whether alterations in brain glucose uptake (BGU), insulin action in the brain–liver axis and whole-body insulin sensitivity occur in young adults in pre-obese state.

**Methods:**

Healthy males with either high risk (HR; *n*  = 19) or low risk (LR; *n*  = 22) for developing obesity were studied with [^18^F]fluoro-d-glucose ([^18^F]FDG)–positron emission tomography during hyperinsulinemic–euglycemic clamp. Obesity risk was assessed according to BMI, physical activity and parental overweight/obesity and type 2 diabetes. Brain, skeletal muscle, brown adipose tissue (BAT), visceral adipose tissue (VAT) and abdominal and femoral s.c. adipose tissue (SAT) glucose uptake (GU) rates were measured. Endogenous glucose production (EGP) was calculated by subtracting the exogenous glucose infusion rate from the rate of disappearance of [^18^F]FDG. BGU was analyzed using statistical parametric mapping, and peripheral tissue activity was determined using Carimas Software imaging processing platform.

**Results:**

BGU was higher in the HR vs LR group and correlated inversely with whole-body insulin sensitivity (M value) in the HR group but not in the LR group. Insulin-suppressed EGP did not differ between the groups but correlated positively with BGU in the whole population, and the correlation was driven by the HR group. Skeletal muscle, BAT, VAT, abdominal and femoral SAT GU were lower in the HR group as compared to the LR group. Muscle GU correlated negatively with BGU in the HR group but not in the LR group.

**Conclusion:**

Increased BGU, alterations in insulin action in the brain–liver axis and decreased whole-body insulin sensitivity occur early in pre-obese state.

## Introduction

Obesity is one of the leading health challenges of the 21st century, resulting in myriad health problems (1). Insulin resistance is a key characteristic of type 2 diabetes (T2D), and alterations in insulin sensitivity may occur even decades before the clinical onset of T2D (2). Obesity is associated with multiorgan insulin resistance (3, 4), impaired suppression of endogenous glucose production (EGP) (5) and adipose tissue lipolysis defined as elevated free fatty acid (FFA) levels (6).

We have previously shown that obese had in spite of their peripheral insulin resistance an increased brain glucose uptake (BGU) during hyperinsulinemic–euglycemic clamp (7), and this finding has been replicated in both humans and animals (8, 9). Large-scale cohort data confirmed that lower peripheral insulin sensitivity is the best predictor of increased BGU during insulin stimulation, and T2D elevates BGU further (10). In line with that, Eriksson and colleagues demonstrated that BGU during insulin clamp was increased in patients with T2D, while the whole-body and peripheral tissue glucose uptake (GU) rates were reduced (11).

Most prior studies evaluating the impact of insulin resistance on BGU have studied middle-aged, obese (BMI > 30 kg/m^2^) or severely obese (BMI > 35 kg/m^2^) individuals, with or without T2D (7, 11, 12). These studies cannot reveal the causality of the increased BGU and insulin sensitivity. It is thus not known whether altered BGU is a consequence of obesity-related metabolic disturbances or whether it can actually represent a further aggravating factor behind these.

BGU correlates positively with EGP in obese but not in lean individuals (5) and also in subjects with a genetic variant associated with impaired intracellular insulin signaling (13), suggesting that in humans, the brain is involved in the control of EGP, but in obese subjects, the insulin action in the brain–liver axis is aberrant. It still remains unresolved whether this is an early characteristic of insulin resistance.

Recently, we have shown that parental overweight and T2D was associated with lower µ-opioid receptor (MOR) and endocannabinoid receptor 1 (CB1R) availability in the brain in overweight but otherwise healthy young males (14). Endogenous opioid system and CB1Rs are involved in the control of food intake and energy homeostasis (15, 16), and previous positron emission tomography (PET) studies have shown downregulation of MOR availability in morbidly obese subjects and normalization after weight loss following bariatric surgery (17). Also, the increased familial obesity risk was associated with increased BGU, while BMI had only a moderate association with BGU (14). However, the association of BGU and insulin sensitivity was not evaluated. The aim of this study is to assess with the same study population, by comparing groups with low vs high obesity risk (LR vs HR), whether alterations in BGU, insulin action in the brain–liver axis and whole-body insulin sensitivity occur already in pre-obese state with risk factors for obesity.

## Subjects and Methods

### Study subjects and study design

We determined the sample size by *a priori* power analysis based on earlier PET studies on obesity (18), suggesting that a sample size of 16 +16 would be sufficient for establishing the predicted effects at *P* < 0.05 with actual power exceeding 0.95, assuming a regional effect size of r = 0.5. We recruited 43 healthy young males with LR (*n*  = 24) or HR (*n*  = 19), as previously described (14). Inclusion criteria for the LR group were male sex, age 20–35 years, BMI 18.5–24.9 kg/m^2^, free time physical activity 4 h or more per week and no maternal or paternal overweight or obesity or T2D. Inclusion criteria for the HR group were male sex, age 20–35 years, BMI 25–30 kg/m^2^, free time physical activity less than 4 h per week and maternal or paternal overweight or obesity or T2D. Overweight, physical inactivity and parental obesity and T2D have been established as risk factors for obesity (19, 20, 21, 22, 23, 24). Exclusion criteria were any chronic disease or medication that would affect glucose metabolism or neurotransmission, history of eating disorder or psychiatric disease, use of nicotine products or abusive use of alcohol. Two LR subjects were excluded because they stopped corresponding. Thus, the study population consisted of 22 LR and 19 HR subjects. One LR subject´s thigh, neck and brain PET scans were not completed according to his wish. Two LR subjects´ brain and one LR subject´s thigh scan were not completed because of scheduling problems.

All subjects underwent a screening consisting of physical examination, anthropometric measurements, fasting blood samples, a 75 g oral glucose tolerance test (OGTT), urine drug-screening test and an inquiry regarding medical history. Body fat mass percentage was measured with an air displacement plethysmograph (the Bod Pod system, software version 5.4.0, COSMED, Inc., Concord, CA, USA) after at least 4 h of fasting. Whole-body magnetic resonance imaging (MRI) and PET studies were performed at the Turku PET Centre (Turku, Finland). The study protocol was approved by the Ethics Committee of the Hospital District of Southwest Finland and conducted in according with the principles of the Declaration of Helsinki. All subjects gave written informed consent prior to inclusion. The study is a part of PROSPECT project registered at ClinicalTrials.gov (Neuromolecular Risk Factors for Obesity, PROSPECT, NCT03106688).

### PET study protocols

We performed the PET studies after a 12-h overnight fast with the GE Discovery (Discovery 690 PET/CT, GE Healthcare) PET camera with [^18^F]fluoro-D-glucose ([^18^F]FDG). Tracer production has been previously described (25). Hyperinsulinemic–euglycemic clamp was applied as previously described (26, 27) in order to measure whole-body insulin sensitivity (Supplementary Text 1). After reaching steady glycemia (80 ± 13 min from the start of the insulin infusion), 156 ± 10 MBq [^18^F]FDG was injected intravenously and dynamic PET scanning was started with the clamp ongoing. Dynamic data of the thoracic region (0–40 min using 4 × 15, 6 × 20, 2 × 60, 2 × 150 and 6 × 300 s frames), upper abdomen (40–55 min; 3 × 300 s) and thighs (55–70 min; 3 × 300 s) and static data of the neck (10 min; 1 × 600 s) and brain (10 min; 1 × 600 s) were collected. Arterialized venous blood samples at 4.5, 7.5, 10, 20 and 30 min from the [^18^F]FDG injection and in the middle time points of the abdomen, thigh, neck and brain scans were taken to measure plasma activity.

### Quantification of tissue glucose uptake

PET data were corrected for dead time, decay and measured photon attenuation before analysis. Input function was quantified by combining image-derived arterial activity data from the left ventricle (28, 29, 30, 31, 32, 33, 34) from 0 to 4.5 min to arterialized plasma sampling taken from 4.5 min to the end of the scan and analyzed with an automatic γ-counter (Wizard 1480 3", Wallac, Turku, Finland). Tissue activity was determined using Carimas Software (version 2.9, Turku PET Centre, downloadable at https://turkupetcentre.fi/software/) and freehand drawing regions of interest (ROI) or volume of interest (VOI) to both quadriceps femoris and hamstrings muscles, the right lobe of the liver, the supraclavicular brown adipose tissue (BAT) depots and several volumes of abdominal s.c. (SAT) and intraperitoneal visceral adipose tissue (VAT). To limit the effect of spillover due to partial volume effect and motion, several ROIs and VOIs in several slices of images were drawn, avoiding large vessels. For myocardium, a segmenting tool of Carimas Software was used to include the left ventricular wall and septum in the analysis. Computed tomography (CT) images were used as references for outlining the regions.

Dynamic tissue time–activity curves and input functions were used to determine the fractional uptake (K_i_) of [^18^F]FDG using the Patlak–Gjedde plot (35) or its approximation fractional uptake rate (FUR) (36). The Patlak–Gjedde plot was used in the analyses of myocardium, liver and femoral skeletal muscle. The mean K_i_ values were extracted from each tissue and used in the analysis. FUR was used in the analyses of VAT, SAT, BAT and brain. Tissue-specific GU (µmol/kg/min) was calculated by multiplying K_i_ or FUR by plasma glucose concentration during scanning and dividing by the tissue density and a lumped constant that accounts for the differences in transport and phosphorylation rates between [^18^F]FDG and glucose. A lumped constant value of 1.2 for skeletal muscle (37, 38), 1.0 for liver (39) and 1.14 for adipose tissue (40) was used. BGU was obtained using an automated processing tool Magia (41) (https://github.com/tkkarjal/magia), as recently described (14). Magia uses FreeSurfer (http://surfer.nmr.mgh.harvard.edu/) to define the ROIs.

### Measurement of endogenous glucose production

EGP during the clamp was determined as previously described (42). Briefly, EGP was calculated by subtracting glucose infusion rate (GIR) from the rate of disappearance of glucose (Rd) during the clamp. Detailed description about the quantification of EGP is described in the Supplementary Text 1.

### Measurement of tissue masses

The abdominal SAT and VAT masses were analyzed from MRI images using sliceOmatic® (Tomovision, Montreal, Quebec, Canada). The femoral SAT and skeletal muscle masses were analyzed from CT images. Detailed description about the tissue mass analyses is provided in the Supplementary Text 3.

### Biochemical analysis

Detailed description about the methods of the biochemical analysis is presented in the Supplementary Text 2.

### Statistical analysis

Data are presented as mean ± s.d. (or median (interquartile range) for non-normally distributed variables). Between-group comparisons were performed with independent samples *t*-test or Wilcoxon rank-sum test as appropriate. Categorical variables were compared with the χ^2^ test. Statistical analyses were performed with SPSS statistical software, version 27. A general linear model and SPM12 (https://www.fil.ion.ucl.ac.uk/spm/software/spm12/) software was used to mode associations between BGU and different predictor variables. The comparison between the groups was performed with a two-sample independent *t*-tests. The statistical threshold in statistical parametric mapping (SPM) analysis was set at a cluster level and corrected with false discovery rate (FDR) with *P* < 0.05. Age was included as a covariate in SPM analysis as age-related brain metabolic changes have been reported (43). Correlations with ROI-specific BGU values and clinical and metabolic variables, BGU were adjusted for each subject to the average age in the whole sample, as previously described (44).

## Results

### Clinical and metabolic characteristics of the study subjects

The HR subjects had less favorable metabolic profile as compared to the LR subjects, comprising of higher weight, BMI, waist-to-hip ratio (WHR), total body fat mass and fat percentage and VAT and abdominal SAT masses. The HR group also had higher blood pressure, worse glycemic and lipid profile, higher values of liver enzymes, high-sensitivity C-reactive protein and glycoprotein acetyls (GlycA) than the LR group. There was a small difference in terms of age between the two study groups (Table 1).

**Table 1:** Anthropometric and metabolic characteristics of the study subjects.

	Low risk (*n* = 22)		High risk (*n* = 19)		*P*
	Mean (s.d.)	Range	Mean (s.d.)	Range	
Age (years)	23 ± 3	20–30	27 ± 4	22–34	0.0005
Weight (kg)	70.3 ± 7.5	57.0–90.9	90.1 ± 9.3	69.4–109.8	<0.0001
BMI (kg/m²)	21.9 ± 2.0	18.5–25.1	27.1 ± 1.9	24.8–31.1	<0.0001
WHR	0.9 ± 0.05	0.8–0.9	0.9 ± 0.05	0.8–1.0	0.002
Body fat mass (kg)^a^	11.7 ± 4.3	5.6–20.6	26.9 ± 9.1	11.4–40.7	<0.0001
Body fat mass (%)^a^	16.4 ± 5.5	7.7–27.9	29.1 ± 7.6	13.9–39.2	<0.0001
Fat-free mass (kg)^a^	59.2 ± 6.6	48.0–76.5	62.8 ± 5.7	51.0–73.3	0.07
Total abdominal VAT (kg)	1.4 ± 0.7	0.3–3.1	3.7 ± 1.2	1.5–5.8	<0.0001
Total abdominal SAT (kg)	2.7 ± 1.0	1.1–5.2	6.8 ± 2.4	2.9–10.4	<0.0001
Systolic blood pressure (mmHg)	123 ± 11	101–145	127 ± 11	117–157	0.001
Diastolic blood pressure (mmHg)	70 ± 10	51–90	74 ± 9	65–101	0.02
Fasting					
Plasma glucose (mmol/L)	4.9 ± 0.5	3.0–5.6	5.5 ± 0.4	4.8–6.3	<0.0001
Plasma insulin (pmol/L)	38.7 ± 21.0	13.9–91.3	61.8 ± 20.8	34.7–97.2	0.0006
Plasma FFA (mmol/L)	0.3 ± 0.1	0.1–0.5	0.3 ± 0.1	0.1–0.7	0.3
2-h OGTT					
Plasma glucose (mmol/L)	4.8 ± 1.0	2.6–6.3	5.9 ± 1.4	3.4–8.2	0.004
Plasma insulin (pmol/L)	160.7 ± 140.1	20.8–694.5	328.2 ± 236.5	34.7–909.8	0.01
Matsuda-ISI	11.0 ± 7.0	3.3–30.6	4.9 ± 2.4	2.1–10.3	0.0008
HbA_1c_(mmol/mol)	29 ± 2	25–35	32 ± 3	27–38	0.004
HbA_1c_ (%)	4.8 ± 0.2	4.4–5.4	5.1 ± 0.3	4.6–5.6	0.007
Total cholesterol (mmol/L)	3.5 ± 0.8	1.8–5.8	4.4 ± 0.9	3.0–6.1	0.002
HDL cholesterol (mmol/L)	1.4 ± 0.3	0.9–1.9	1.3 ± 0.3	0.9–2.1	0.2
LDL cholesterol (mmol/L)	2.1 ± 0.8	0.4–3.7	2.9 ± 0.8	1.8–4.1	0.0009
Triglycerides (mmol/L)	0.7 ± 0.3	0.3–1.4	1.2 ± 0.6	0.3–2.9	0.004
ApoB (g/L)	0.6 ± 0.1	0.4–0.7	0.8 ± 0.2	0.6–1.3	<0.0001
ApoA1 (g/L)	1.3 ± 0.2	1.1–1.6	1.3 ± 0.1	1.1–1.6	0.8
ApoB/ApoA1	0.5 ± 0.1	0.3–0.7	0.6 ± 0.1	0.4–1.0	0.0001
ALT (U/L)	22 ± 8	14–43	32 ± 10	13–49	0.001
AFOS	70 ± 17	50–119	60 ± 19	35–100	0.08
GGT (U/L)	15 ± 5	8–27	33 ± 28	6–124	0.008
hs-CRP (mg/L)	0.5 ± 0.5	0.1–2.4	1.1 ± 1.1	0.1–4.6	0.01
GlycA (mmol/L)	0.7 ± 0.06	0.6–0.8	0.8 ± 0.06	0.7–0.9	<0.0001
Steady-state glucose (mmol/L)	5.3 ± 0.2	4.6–5.9	5.3 ± 0.3	4.8–5.6	0.5
Steady-state insulin (ρmol/L)	510.5 ± 76.8	349.6–632.0	581.7 ± 94.9	476.4–801.5	0.01
M value (µmol/kg/min)	58.0 ± 14.7	30.2–86.1	38.7 ± 13.7	17.4–72.3	0.0001
M value (µmol/kg_FFM_/min)	69.6 ± 17.3	35.2–102.3	53.0 ± 17.1	24.1–100.7	0.004
EGP (µmol/kg/min)^b^	–3.4 ± 6.7	–13.5 to 10.5	0.9 ± 6.0	–13.5 to 7.9	0.2
EGP (µmol/kg_FFM_/min)^b^	–2.2 ± 8.9	–19.2 to 13.2	–0.6 ± 8.9	–16.1 to 11.7	0.6
Clamp FFA (mmol/L)	0.03 ± 0.01	0.01–0.1	0.05 ± 0.03	0.02–0.1	0.03

^a^Body fat mass (kg and %) for high-risk subjects is calculated with *n*  = 18, since one high-risk subject did not complete the body composition measurement.

^b^EGP for low-risk subjects is calculated with *n*  = 21, since one low-risk subject did not feel the need to urinate.

AFOS, alkaline phosphatase; ALT, alanine transaminase; ApoB, apolipoprotein B; ApoA1, apolipoprotein A1; BMI, body mass index; EGP, endogenous glucose production; FFA, free fatty acids; FFM, fat-free mass; GlycA, gGlycoprotein acetyls; GGT, gamma-glutamyltransferase; hs-CRP, high-sensitivity C-reactive protein; Matsuda-ISI, insulin sensitivity index by Matsuda; OGTT, oral glucose tolerance test; SAT, s.c.. adipose tissue; VAT, visceral adipose tissue; WHR, waist-to-hip ratio.

### Risk group differences in the whole-body insulin sensitivity

During the clamp, plasma glucose levels were steady at 5.3 mmol/L in both groups. The HR group had higher steady-state insulin levels and smaller suppression of FFA as compared to the LR group (Table 1 and Supplementary Fig. 1, see section on supplementary materials given at the end of this article), and these two measurements were reciprocally related (r = –0.41, *P* = 0.009).

Whole-body insulin sensitivity indexed by the M value and the whole-body Rd were significantly lower in the HR group than in the LR group. Insulin-suppressed EGP did not significantly differ between the groups (Table 1). In the whole dataset, M value and Rd correlated negatively with FFA levels during the clamp, and the correlation was driven by the HR group. Furthermore, M value correlated positively with Rd and negatively with EGP in both groups and in the whole dataset. M value correlated negatively with VAT and abdominal SAT masses (Fig. 2), while no association was found between EGP and adipose tissue masses.

**Figure 1:**
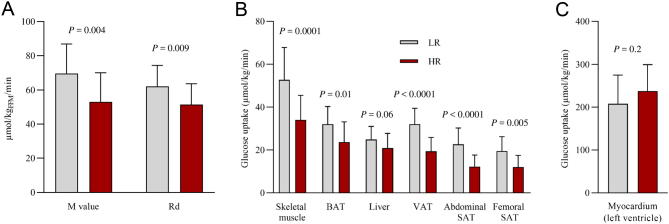
Whole-body and tissue-specific glucose uptake rates. (A) Whole-body insulin sensitivity indexed by the M value and whole-body glucose disposal rate (Rd) calculated with the fat-free mass in the low-risk (LR) and in the high-risk (HR) group. (B) Glucose uptake rates in skeletal muscle, brown adipose tissue (BAT), liver, visceral adipose tissue (VAT) and abdominal and femoral s.c. adipose tissue (SAT) and (C) myocardium (left ventricle) in the LR and in the HR group. Bar heights represent sample means. Vertical lines represent sample s.d. *P* values for comparison of LR vs HR group.

**Figure 2:**
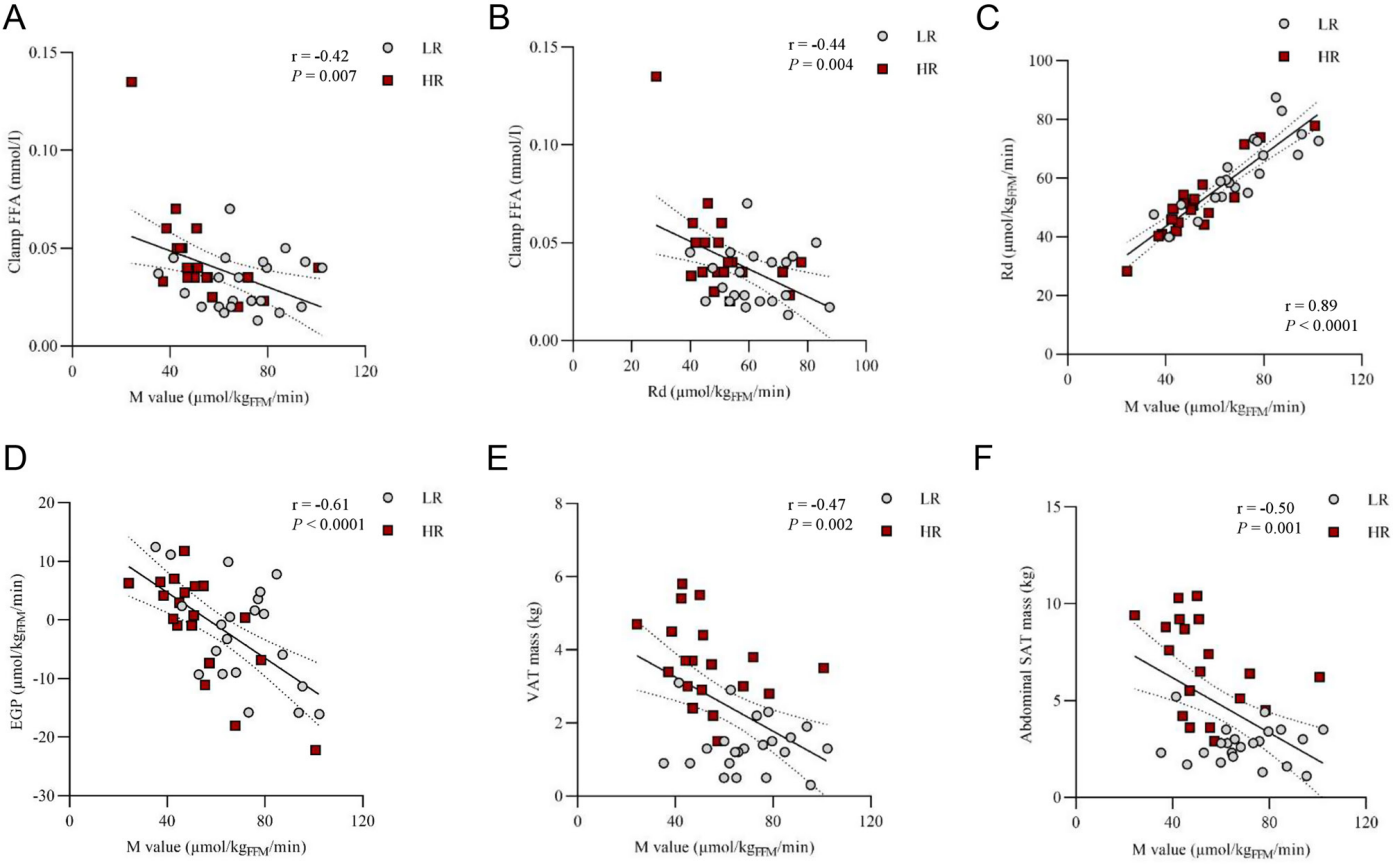
Association between the whole-body insulin sensitivity indexes and adipose tissue masses. (A) Association between the M value and free fatty acid (FFA) levels and (B) Rd and FFA levels during the hyperinsulinemic–euclycemic clamp, (C) M value and the rate of disappearance (Rd), (D) M value and endogenous glucose production (EGP), (E) M value and visceral adipose tissue (VAT) mass, and (F) M value and abdominal s.c. adipose tissue (SAT) mass.

**Figure 3:**
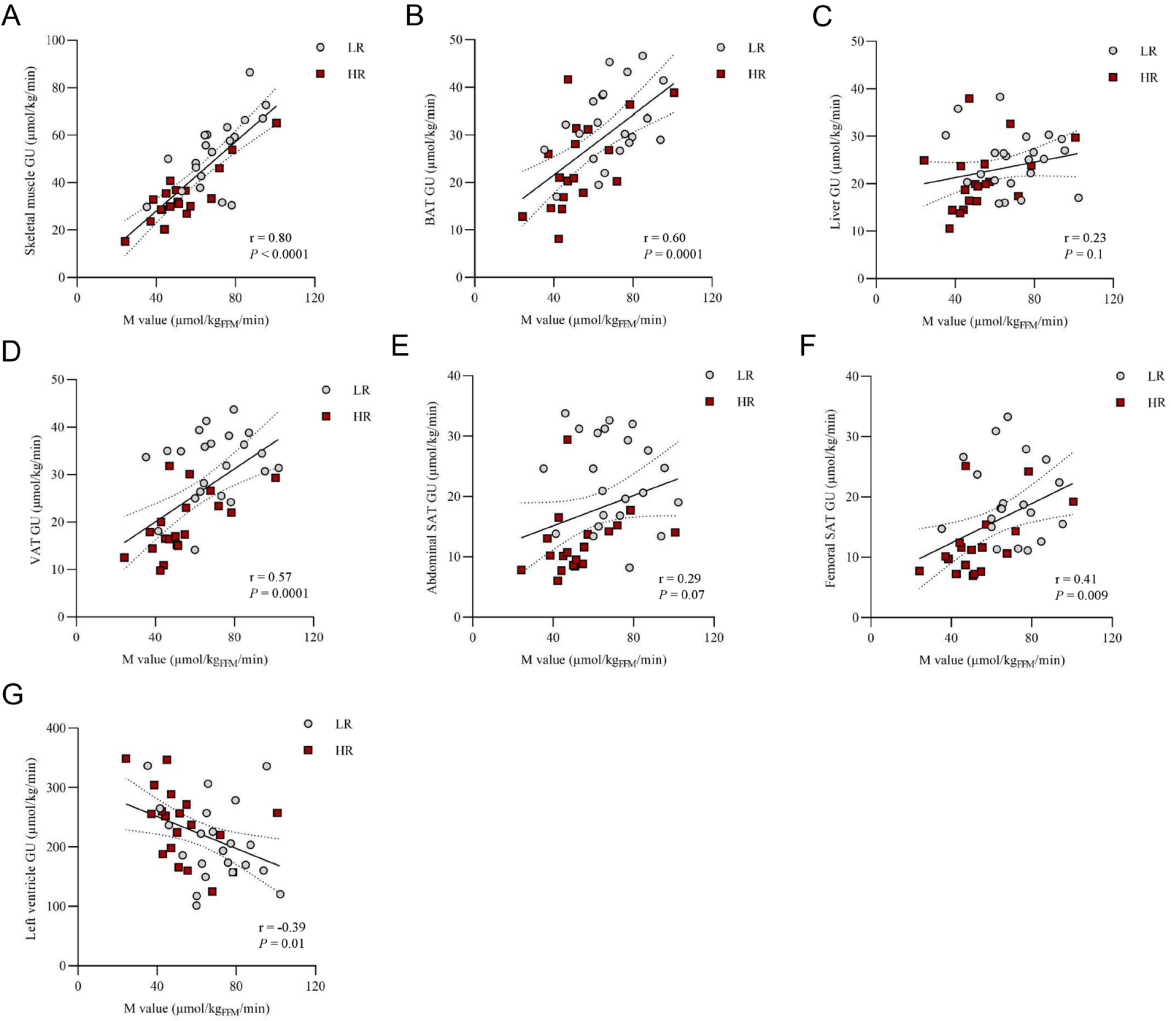
Association between the whole-body insulin sensitivity (M value) and the tissue-specific glucose uptake rates (GU) in (A) femoral skeletal muscle, (B) brown adipose tissue (BAT), (C) liver, (D) visceral adipose tissue (VAT), (E) abdominal s.c. adipose tissue (SAT), (F) femoral SAT and (G) myocardium left ventricle in the low-risk (LR) and in the high-risk (HR) group.

The HR group was characterized by a multi-tissue insulin resistance, as demonstrated by lower rates of GU in skeletal muscle, liver, VAT, abdominal and femoral SAT and BAT (Fig. 1B). In line with this, the M value correlated positively with the GU rate in the skeletal muscle, BAT, VAT and femoral SAT, and the associations were driven by the HR group, but no association was found between the M value and liver or abdominal SAT GU (Fig. 3 and Supplementary Table 2). Myocardial left ventricle GU rates were not different between groups (Fig. 1C) and correlated negatively with the M value (Fig. 3G and Supplementary Table 2).

When calculated the depot-specific GU, we found a lower rate of GU in the skeletal muscle in the HR group than in the LR group, but higher rates of GU in the VAT and abdominal SAT because of the fat mass expansion in the HR group. The depot-specific GU in the femoral SAT did not significantly differ between the groups, although the HR subjects had higher proportion of fat in the analyzed femoral section as compared to the LR group (Table 2).

**Table 2:** Tissue masses and tissue depot-specific insulin-stimulated glucose uptake (GU) rates in the low-risk (LR) and in the high-risk (HR) group. Data presented as mean ± s.d.

	Tissue mass (kg)		P*	Depot-specific GU (µmol/min)		P**
	LR	HR		LR	HR	
Brain	1.7 ± 0.2	1.7 ± 0.1	0.5	258.1 ± 46.2	270.2 ± 43.2	0.4
Femoral skeletal muscle^a^	5.6 ± 0.6	5.8 ± 0.5	0.1	292.5 ± 80.3	199.9 ± 75.5	0.0007
VAT	1.4 ± 0.7	3.7 ± 1.2	<0.0001	42.1 ± 18.4	68.8 ± 27.0	0.0006
Abdominal SAT	2.7 ± 1.0	6.8 ± 2.4	<0.0001	52.2 ± 23.3	79.7 ± 34.8	0.02
Femoral SAT	2.1 ± 0.7	2.8 ± 0.8	0.01	40.0 ± 19.2	31.7 ± 14.6	0.1

^a^Covering the 15 cm section of the mid-thigh in both groups

*P value is for two-tailed independent samples *t*-test for tissue mass between the two groups.

**P value is for two-tailed independent samples *t*-test for depot-specific GU between the two groups.

SAT, s.c. adipose tissue; VAT, visceral adipose tissue.

### Increased BGU in the high-risk group

**Figure 4:**
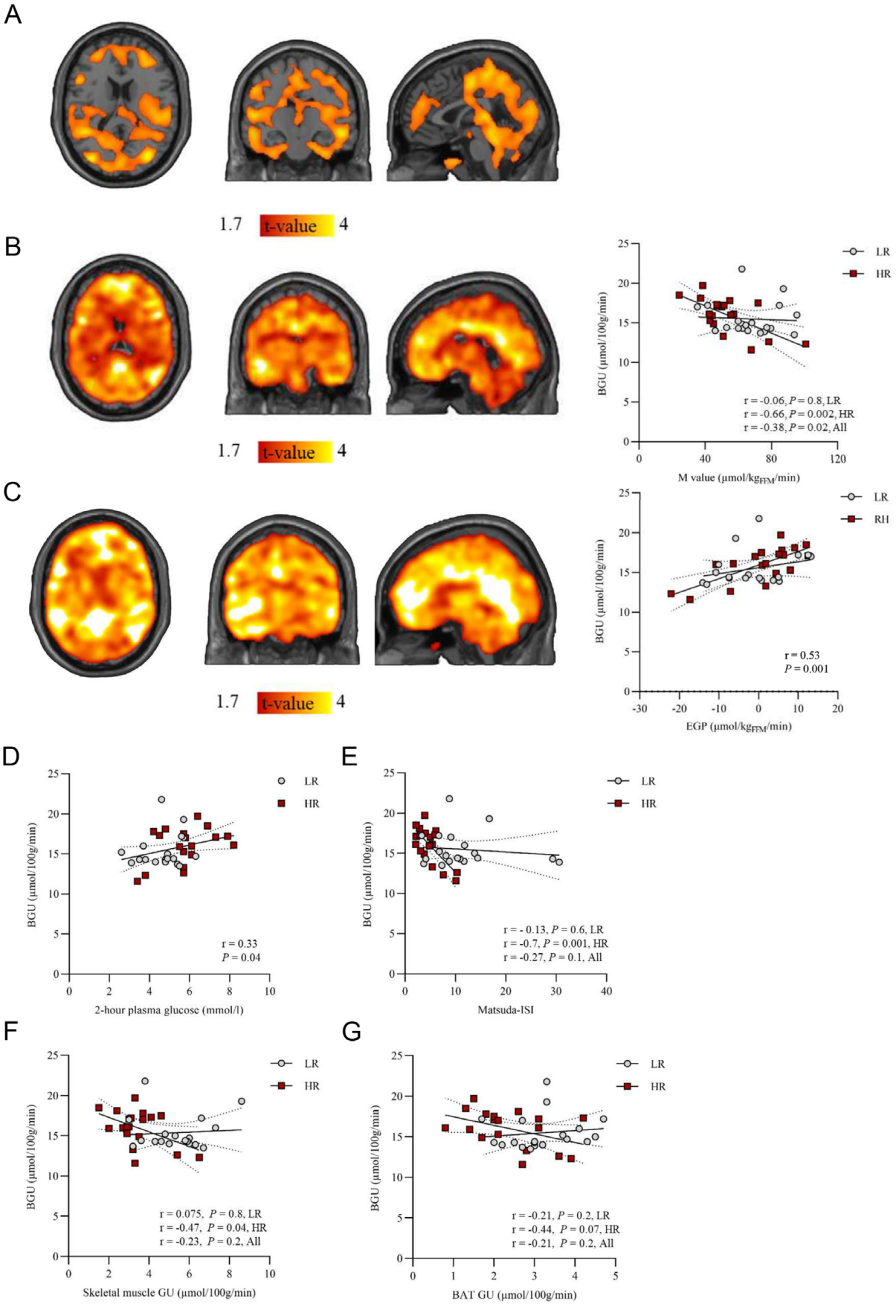
Brain glucose uptake (BGU) in the high-risk (HR) and the low-risk (LR) group. (A) A statistical parametric mapping (SPM) two-sample *t*-test between the HR and the LR group. Marked brain areas show regions with significantly higher insulin-stimulated BGU in the HR group as compared to the LR group. Higher T values denote larger differences between the groups. *P* values <0.05 at a cluster level and false discovery rate (FDR) corrected. (B) Brain clusters (as defined by FDR-corrected SPM one-sample *t*-test) for the association between BGU and M value and (C) BGU and EGP in the whole study group and the corresponding scatterplots. For the scatterplots, global region of interest (ROI) was extracted and used. (D) Association between the BGU and 2-h plasma glucose level in oral glucose tolerance test, (E) BGU and insulin sensitivity index by Matsuda (Matsuda-ISI), (F) BGU and skeletal muscle glucose uptake (GU) and (G) BGU and brown adipose tissue GU in the LR group and in the HR group.

At the voxel-by-voxel level, insulin-stimulated BGU was higher in the HR group as compared to the LR group (Fig. 4A), as reported previously (14). Cross-sectionally, the M value correlated negatively with BGU, but the correlation was driven by the HR group (Fig. 4B and Supplementary Table 2). Insulin-stimulated BGU correlated positively with insulin-suppressed EGP in the whole dataset (Fig 4C), and the association was driven by the HR subjects (Supplementary Tables 2–3).

FFA levels during the clamp correlated positively with BGU in the ROI level in the whole dataset, and the association was driven by the HR group (Supplementary Table 3).

Insulin-stimulated BGU correlated positively with 2-h plasma glucose level in OGTT among all study subjects and negatively with Matsuda-ISI in the HR group but not in the LR group (Fig. 4D, E).

### Inter-tissue associations between the insulin sensitivity and body composition

BGU in the HR group correlated negatively with skeletal muscle GU and showed a trend toward a negative correlation with BAT GU (Fig. 4F, G), whereas no correlation was found in the LR group. In both HR and LR groups, we did not find an association between BGU and liver, abdominal SAT, VAT, or femoral SAT GU rates, but we did find a positive association between BGU and myocardial left ventricle GU (Supplementary Table 1). In the whole dataset, BGU correlated positively with several measures of adiposity comprising VAT and abdominal SAT masses, which were mainly driven by the HR group (Supplementary Table 4).

WHR and total body fat percentage were positively associated with BGU at the ROI level in the HR group. In the LR group, there was a tendency of negative association between WHR and BGU and no association between total body fat percentage and BGU (Supplementary Table 4).

Inter-tissue associations between GU rates in brain and peripheral tissues are presented in Supplementary Table 1.

Furthermore, GU rates in skeletal muscle, BAT, and abdominal and femoral SAT, but not BGU, correlated negatively with apolipoprotein B (ApoB), apolipoprotein A1 (ApoA1) and ApoB/ApoA1 ratio, total and LDL cholesterol, triglycerides and inflammatory marker GlycA (Supplementary Table 2).

## Discussion

Our main finding based on the risk-group comparison was that both an increased BGU during hyperinsulinemic–euglycemic clamp and a defective insulin action in the brain–liver axis are early features of insulin resistance, being present already in healthy pre-obese subjects with increased adiposity, in early adulthood. These results accord with prior studies showing that during euglycemic hyperinsulinemia BGU is increased in conditions with systemic insulin resistance (8, 10, 11, 13). Even though the molecular mechanisms of this phenomenon are not known, our study demonstrates that relatively mild alterations in metabolic regulation are sufficient for this change to occur. Indeed, the inclusion criterion to the HR group was obesity risk in the early adulthood.

The HR group was characterized by impaired systemic insulin sensitivity. Compared with the LR group, they had reduced whole-body and tissue-specific GU rates and impaired suppression of adipose tissue lipolysis during the clamp. EGP, however, did not significantly differ between the groups. The HR subjects had worse metabolic profile based on general risk factors such as blood pressure, lipid values, OGTT, WHR and body adiposity, and coherently there were significant associations between these and the clamp-derived measurements (Supplementary Table 2). Considering this and the increased insulin-stimulated BGU found in the HR as compared to the LR group, our results are converging with the previous findings in obese subjects (7) and subjects with impaired glucose tolerance (8, 12).

There is long-standing debate regarding when and where insulin resistance starts, with some researchers proposing an important role of the brain in the regulation of energy balance and glucose homeostasis (45). Taking together both the enhanced BGU and the impaired insulin action in the brain–liver axis already in early adulthood, our data further contribute to the increasing understanding that brain metabolism is linked to impairments of whole-body homeostasis.

It is now well-established that accumulation of fat in the femoral SAT may play a protective metabolic role (46, 47), whereas expansion of fat mass in the abdominal SAT and VAT is associated with metabolic abnormalities such as insulin resistance, hyperglycemia, hypertension, and dyslipidemia (48). In a recent study, it was shown that subjects with strong insulin-induced suppression in hypothalamic blood flow (measured with functional MRI) had significantly less VAT, but there was no association between central insulin action and SAT (49); there was no distinction between abdominal and femoral SAT. Interestingly, in the present dataset, insulin-stimulated BGU correlated positively both with VAT and with abdominal SAT mass but not with femoral SAT mass. Further, we found that a significantly higher proportion of whole-body glucose metabolism during hyperinsulinemia occurs in VAT (*P* = 0.0008) and abdominal SAT (*P* = 0.02) in the HR subjects as compared to the LR subjects. Despite the correlational nature of our data which cannot explain the basis of this differential pattern of association between BGU and fat masses in different fat depots, this could be attributed to the differences in endocrine function (e.g. adipokine secretion and FFA metabolism) and neural innervation between the various fat depots (50, 51, 52).

We have previously shown that in a group of morbidly obese individuals studied before and 6 months after bariatric surgery, a higher BGU at baseline predicted worse glycemic control at 2 and 3 years of follow-up (5). Also, in a similar group studied with a fatty acid PET tracer 14(R,S)-[^18^F]fluoro-6-thia-heptadecanoic acid, enhanced brain fatty acid uptake at baseline predicted worse glycemic control following bariatric surgery (53). These findings would be essentially in line with the results of a previous intervention study (TULIP) where decreased cerebrocortical response to insulin in magnetoencephalography (defined by the authors as central insulin resistance) predicted a worse adhesion to the diet intervention and less weight loss (54). In this PROSPECT study design, we will further assess whether the BGU at baseline will predict weight and glycemic status at 5-year follow-up. This differs from the bariatric surgery setting, where significant weight loss occurs rapidly.

Strengths of the present study are the strict selection of the study participants after a thorough evaluation of their obesity risk factors, adiposity, and the application of PET, which not only represents the state-of-the art method for the assessment of glucose metabolic rates *in vivo* but also enables the contemporaneous assessment of EGP. Our study has certain limitations. First, we studied only males, and whether the results can be extrapolated to women is not known. In the previous large-scale study, BGU under insulin stimulation was not significantly affected by sex (10), and therefore it is possible that the effect we found could be similar with women. Subjects in the HR group were somewhat older than those in the LR group, and age represents an important determinant of BGU in both fasting and clamp conditions (55). To circumvent this problem, age was used as a covariate in the BGU analyses, and the results were unchanged. Unfortunately, one LR subject had BMI 25.1 kg/m² and one HR subject 24.8 kg/m² although otherwise fulfilling the group criteria. For the consistency of the statistics, we decided not to exclude them. EGP values in both study groups were mainly negative, indicating EGP suppression under insulin stimulation. A previous study showed a progressive decline in insulin suppression of EGP with increasing VAT mass and hepatic lipid content (HLC) (56). We did not measure HLC in the this study; however, in a recent study, 95% percentile of HLC of lean non-Asian Indian participants was shown to be 1.85% (57), considerably lower than the previously determined upper limit of normal HLC 5.56% (58). Thus, it can be assumed that in the present study where the LR subjects are lean and the HR subjects are only overweight, not obese, it is likely that most of the subjects would have HLC under 1.85% and have effective suppression of EGP. Also, large variability and negative EGP values has been shown also in previous studies (59).

In conclusion, this risk-group comparison demonstrates that an increased BGU and a dysregulated insulin action in the brain–liver axis occur already in the pre-obese state in young healthy subjects, in the age range of early adulthood and with only mild extra adiposity. This cross-sectional study cannot resolve the causality of the effect, and longitudinal and intervention studies are needed to address this. The follow-up period may potentially show whether the enhanced BGU has an impact on metabolic deterioration with advancing age.

## Supplementary Material

Supplementary Material

Supplementary Figure 1

## Declaration of interest

The authors declare that there is no conflict of interest that could be perceived as prejudicing the impartiality of the research reported.

## Funding

The study was supported by Center of Excellence funding (no. 307402) to PN, Academy of Finland http://dx.doi.org/10.13039/501100002341 grant nos 294897 and 332225 to LN, Jalmari and Rauha Ahokas Foundation, Turunmaa Duodecim Society, Turku University Hospital Foundation for Education and Research and The Diabetes Research Foundation to LP, Finnish Cultural Foundation (Southwest Finland Fund), Emil Aaltonen Foundation and Jenny and Antti Wihuri Foundation to TK, Finnish Cultural Foundation and Maud Kuistila Memorial Foundation to ER, and Sigrid Juselius Foundation.

## Data availability

The data from this study are available from the corresponding author PN on reasonable request.

## Author contribution statement

LP: first author, study design, study coordination, data acquisition, data modeling, statistical analysis, interpretation of the results, tables and figures and main writer of the manuscript. TK: study design, study coordination, data acquisition, data modeling, statistical analysis, interpretation of the results and writing of the manuscript. ER: brain PET data modeling, statistical analysis, interpretation of the results, figures and writing of the manuscript. AL-R and PD: participation in the PET data analysis and writing of the manuscript. ToK and MB: brain PET data modeling. KK: study design, especially effects of exercise on metabolism. AKK: radiotracer production. KL and NH: measurement of the body fat percentage. TR: study design, expertise in metabolic risk factors and writing of the manuscript. LN: study design, study coordination, interpretation of the results, writing of the manuscript and supervision of the study. All authors approved the final version of the manuscript. PN: corresponding author, study design, study coordination, interpretation of the results, writing of the manuscript and supervision of the study. As a guarantor of the work has full access to all the data in the study and takes responsibility for the integrity of the data analysis.
