# Supplementary material for: Obesity risk is associated with brain glucose uptake and insulin resistance
Source: Eur J Endocrinol. 2022 Oct 26;187(6):917–28. doi: 10.1530/EJE-22-0509 (PMC9782452; doi:10.1530/EJE-22-0509)
Supplement: Supplementary Material [file supplementary_material.pdf]

SUPPLEMENTARY MATERIALS

I. Hyperinsulinemic-euglycemic clamp

II. Measurement of Endogenous Glucose Production

III. Measurement of Tissue Masses

IV. Biochemical Analysis

V. Supplementary Tables

VI. Legends to Supplementary Figures

VII. Refecences

HYPERINSULINEMIC-EUGLYCEMIC CLAMP

Supplementary Text 1

Subjects were in a supine position and two catheters were inserted in veins of opposite forearms, one in an antecubital vein for insulin and glucose infusion and for radiotracer injection and one in the contralateral antecubital vein for blood sampling. To obtain arterialized venous blood samples, the arm was warmed with a heating pillow. After collecting fasting laboratory samples, a hyperinsulinemic-euglycemic clamp was started. Insulin (Actrapid, Novo Nordisk A/S, Bagsvaerd, Denmark) was given in a primed-continuous manner at the rate of 40 mU/m^2 body surface area/min. Plasma glucose measurements were taken every 5 to 10 minutes during the study, and 20 % glucose solution was infused at a variable rate to maintain euglycemia, (5.0 ± 0.5 mmol/l). Whole-body insulin sensitivity was indexed by the M value, calculated as the average of 20-min intervals between 60-160 min after the start of insulin infusion.

MEASUREMENT OF ENDOGENOUS GLUCOSE PRODUCTION

Supplementary Text 2

Endogenous glucose production (EGP) during the hyperinsulinemic-euglycemic clamp was calculated by subtracting glucose infusion rate (GIR) from the rate of disappearance of glucose (Rd) during the clamp with the following formula: EGP = Rd + $V_{glucose}$ x $\Delta_{glucose}/\Delta_T$ – GIR. GIR is corrected by a space correction [1], that accounts the possible changes in glucose level in plasma and interstitial over the study. $V_{glucose}$ is an estimated constant for glucose distribution volume (0.19 l/kg), $\Delta_{glucose}$ is the change in glucose level from [^{18}F]FDG injection to the end of sampling (mmol/l), Δ_T is the time of [^{18}F]FDG injection to the end of sampling (min) and GIR is the total amount of infused glucose during the scan (mg/kg). R_d (µmol/min/kg) was calculated using the equation: R_d = MCR_{FDG} x $avg_{glucose}$ /weight, where MCR_{FDG} (ml/min) is the metabolic clearance rate of [^{18}F]FDG, avg $_{glucose}$ is the average plasma glucose level (mmol/l) from the time of [^{18}F]FDG injection to the

end of sampling, and weight is the subject´s weight (kg). MCR_{FDG} was calculated with the following formula: MCR_{FDG} = ($dose_{FDG}$ –$urine_{FDG}$)/AUC_{FDG}, where $dose_{FDG}$ is the injected [^{18}F]FDG dose (kBq), $urine_{FDG}$ is the amount of tracer lost to urine during the entire scan (kBq), and AUC_{FDG} is the area under the curve of [^{18}F]FDG in plasma from [^{18}F]FDG injection to the end of sampling. The amount of [^{18}F]FDG lost into urine were measured with an isotope dose calibrator (Model VDC-205; Comecer Netherlands, Joure, Netherlands).

MEASUREMENT OF TISSUE MASSES

Supplementary Text 3

The abdominal subcutaneous (SAT) and visceral (VAT) adipose tissue mass were analyzed from MRI images using sliceOmatic® (Tomovision, Montreal, Quebec, Canada). A whole-body magnetic resonance imaging was performed at 3T after a 12-hour fasting using the MRI part of a clinical PET-MRI system (Philips Ingenuity TF PET/MR, Philips, Amsterdam, Netherlands). The abdominal SAT compartment was defined as the fat depot between the skin and the above the abdominal musculature3. The abdominal VAT mass was measured from the combination of the intraperitoneal and retroperitoneal fat compartments [2, 3].

The femoral SAT and skeletal muscle mass were analyzed from CT images because of artefacts in the MRI images in the femoral region. The CT images were acquired with PET/CT (GE Discovery VCT PET/CT, GE Healthcare) while performing the femoral skeletal muscle GU measurement during the clamp. The femoral SAT and skeletal muscle mass analysis were performed with Carimas version 2.9 (http://turkupetcentre.fi/carimas/download). A total of 47 slices of CT-derived images covering the length of 15 cm, the height of the scanned femoral area, in the mid-section of the thighs of both lower limbs were used for the analysis. That is, the quantified femoral SAT and skeletal muscle masses did not cover the entire femoral SAT and muscle mass, but the analysis were done similarly with every subject. The attenuation threshold value of -300 to -10

Hounsfield units (HU) was used to define the adipose tissue regions[4] and between -29 HU to +150 HU was used for skeletal muscle [5]. The MRI and CT derived tissue volumes were converted to their respective mass (kg) by assuming tissue densities of 0.9196 kg/l for adipose [6] and 1.0597 kg/l for skeletal muscle [7]. Brain volumes were determined from MRI images and converted to their respective mass using a density value of 1.04 kg/l [8].

BIOCHEMICAL ANALYSIS

Supplementary Text 4

Plasma glucose concentrations during the hyperinsulinemic-euglycemic clamp were determined in duplicates using the glucose oxidate method (Analox GM9; Analox Instruments, London, UK). Plasma insulin concentrations were analyzed using an automated electrochemiluminescence immunoassay (Cobas 8000; Roche Diagnostics) and serum free fatty acid (FFA) concentrations with an enzymatic colorimetric method (NEFA-HR2, ACS-ACOD; Wako Chemicals, Neuss, Germany; Cobas 8000 c502 and Cobas 800 c702 Analyzer, Roche Diagnostics). Plasma glucose values at fasting and in oral glucose tolerance test (OGTT) were determined using an enzymatic photometry/hexokinase reaction (Cobas 8000 c 702; Roche Diagnostics) and HbA_{1c} with immunoturbidimetry (Cobas 6000 c 501, Roche Diagnostics). Total plasma cholesterol and HDL and LDL cholesterol were measured with a direct photometric enzymatic assay (Cobas 8000 c 702, Roche Diagnostics) and plasma triglycerides with a photometric enzymatic assay (GPO-PAP; Cobas 8000 c 702, Roche Diagnostics). Plasma creatinine was measured with a photometric enzymatic assay (Cobas 8000 c 702, Roche Diagnostics) and serum high-sensitivity C-reactive protein (hs-CRP) with immunonefelometry (BN ProSpec System; Siemens Healthineers). Plasma alanine aminotransferase, alkaline phosphatase and gamma-glutamyltransferase (GGT) in fasting state were assessed using a kinetic photometry according to IFCC recommendation (Cobas 8000 c 702, Roche Diagnostics). Glycoprotein acetyls, apolipoprotein B (ApoB), apolipoprotein A1 (ApoA1) and ApoB/ApoA1 were meas-

ured as a part of metabolic biomarker panel that was quantified from plasma samples using high-throughput proton nuclear magnetic resonance (NMR) [9] metabolomics (Nightingale Health Oyj, Helsinki, Finland).

SUPPLEMENTARY TABLES

Table 1 Inter-tissue associations between insulin-stimulated glucose uptake rates in brain, liver, femoral skeletal muscle, brown adipose tissue (BAT), visceral adipose tissue (VAT), abdominal and femoral subcutaneous adipose tissue (SAT) and myocardium left ventricle. Data are Pearson´s r values. Significant associations are indicates by bold font. * $P < 0.05$, ** $P < 0.001$.

	Insulin-stimulated glucose uptake rate (µmol/kg/min)							
	Brain	Liver	Femoral skeletal muscle	BAT	VAT	Abdominal SAT	Femoral SAT	Left ventricle
Brain		-0.27	-0.22	-0.21	-0.15	-0.06	-0.06	**0.39***
Liver	-0.27		**0.45****	0.22	**0.36****	0.27	0.25	-0.03
Femoral skeletal muscle	-0.22	**0.45****		**0.62****	**0.66****	**0.54****	**0.58****	-0.14
BAT	-0.21	0.22	**0.62****		**0.64****	**0.61****	**0.64****	-0.27
VAT	-0.15	**0.36***	**0.66****	**0.64****		**0.81****	**0.73****	-0.05
Abdominal SAT	-0.06	0.27	**0.54****	**0.61****	**0.81****		**0.80****	0.02
Femoral SAT	-0.06	0.25	**0.58****	**0.64****	**0.73****	**0.80****		-0.09
Left ventricle	**0.39***	-0.03	-0.14	-0.27	-0.05	0.02	-0.09	

	Insulin-stimulated glucose uptake rate (µmol/kg/min)

Table 2 Associations between insulin-stimulated glucose uptake rate in brain, liver, femoral skeletal muscle, brown adipose tissue (BAT), visceral adipose tissue (VAT), abdominal and femoral subcutaneous adipose tissue (SAT) and myocardium left ventricle and anthropometric and metabolic characters in the whole study group. Data are Pearson´s r values. Significant associations are indicates by bold font. * $P < 0.05$, ** $P < 0.001$.

	Brain	Liver	Femoral skeletal muscle	BAT	VAT	Abdominal SAT	Femoral SAT	Left ventricle
Age (years)	-0.18	0.03	**-0.49****	**-0.33***	**-0.38***	**-0.36***	**-0.36***	0.05
Weight (kg)								
BMI (kg/m^2)	0.11	-0.21	**-0.52****	**-0.49****	**-0.64****	**-0.64****	**-0.59****	0.14
WHR	0.09	-0.26	**-0.46****	**-0.52****	**-0.59****	**-0.59****	**-0.44****	-0.13
Body fat mass (kg)	0.24	-0.31	**-0.55****	**-0.68****	**-0.72****	**-0.69**	**-0.68**	0.26
Body fat mass (%)	0.21	-0.25	**-0.58****	**-0.71****	**-0.72****	**-0.74****	**-0.71****	0.26
Fat-free mass (kg)	-0.06	-0.06	-0.12	0.07	-0.08	0.12	0.08	-0.06
VAT mass (kg)	0.18	-0.20	**-0.56****	**-0.69****	**-0.75****	**-0-70****	**-0.64****	0.19
Abdominal SAT mass (kg)	0.21	**-0.35***	**-0.56****	**-0.63****	**-0.72****	**-0.68****	**-0.64****	0.26
Systolic BP (mmHg)	0.12	-0.08	**-0.39***	**-0.44****	**-0.42****	**-0.45****	**-0.52****	0.26
Fasting plasma glucose (mmol/l)	0.14	**-0.48****	**-0.51****	-0.24	**-0.57****	**-0.48****	**-0.38***	0.15
2-hour plasma glucose (mmol/l)	**0.33***	**-0.34***	**-0.42****	**-0.38***	**-0.40***	**-0.45****	**-0.35***	0.19
HbA1c (mmol/mol)	0.09	-0.16	-0.28	-0.06	**-0.44****	-0.23	-0.24	0.11
Matsuda-ISI	-0.27	**0.48****	**0.52****	0.31	**0.51****	**0.42****	**0.42****	-0.19
Total cholesterol (mmol/l)	-0.04	0.04	**-0.36***	**-0.40***	**-0.40****	**-0.34***	-0.29	0.05
LDL cholesterol (mmol/l)	-0.03	0.005	**-0.41****	**-0.46**	**-0.52****	**-0.46****	**-0.39***	0.02
Triglycerides (mmol/l)	0.10	-0.29	**-0.45****	-0.31	**-0.42****	**-0.35***	-0.31	0.03
ApoB (g/l)	0.08	-0.09	**-0.53****	**-0.44****	**-0.55****	**-0.45****	**-0.48****	0.17
ApoA1 (g/l)	-0.06	0.27	0.01	0.30	**0.37***	**0.47****	0.31	0.02
ApoB/ApoA1	0.15	-0.21	**-0.51****	**-0.56****	**-0.67**	**-0.61****	**-0.56****	0.15
GGT (U/l)	0.25	-0.04	**-0.34***	-0.16	**-0.36***	-0.31	**-0.34***	0.17
hs-CRP (mg/l)	0.20	-0.18	-0.0002	-0.28	-0.23	-0.22	-0.19	-0.04
GlycA (mmol/l)	0.15	-0.14	**-0.43****	**-0.45****	**-0.52****	**-0.54****	**-0.42***	0.11
M value (µmol/kg/min)	**-0.33***	0.28	**0.85****	**0.67****	**0.65****	**0.44****	**0.53****	**-0.36***
M value (µmol/kg$_{FFM}$/min)	**-0.38***	0.23	**0.80****	**0.60****	**0.57****	0.29	**0.41****	**-0-40***
EGP (µmol/kg/min)	**0.48***	-0.14	-0.24	-0.20	-0.18	0.02	-0.21	**0.43***
EGP (µmol/kg$_{FFM}$/min)	**0.53****	-0.14	-0.29	-0.24	-0.23	-0.08	-0.25	**0.41***
Steady-state insulin (ρmol/l)	0.10	-0.24	-0.31	**-0.39***	**-0.41****	**-0.35***	**-0.39***	0.23
Steady-state FFA (mmol/l)	0.27	-0.06	**-0.33***	**-0.44****	**-0.51****	**-0.38***	**-0.40***	**0.38***

Abbreviations: ApoB, apolipoprotein; ApoA1, apolipoprotein A1; BMI, body mass index; BP, blood pressure; EGP, endogenous glucose production; FFA, free fatty acids; FFM, fat-free mass; GlycA, Glycoprotein acetyls; GGT, gamma-glutamyltransferase; hs-CRP, high-sensitivity C-reactive protein; Matsuda-ISI, insulin sensitivity index by Matsuda; WHR, waist-to-hip ratio.

Table 3 Associations between the insulin-stimulated brain glucose uptake rates at the ROI level and the whole-body glucose uptake (M value), insulin-suppressed endogenous glucose production (EGP) and steady-state free fatty acid (FFA) levels among the all study subjects, in the low-risk (LR) and in the high-risk (HR) group. M and EGP values calculated with the fat-free mass were used in the analysis. Data are Pearson´s r values. Significant associations are indicates by bold font. * $P < 0.05$, ** $P < 0.001$.

	M value			EGP			FFA		
	All	LR	HR	All	LR	HR	All	LR	HR
Amygdala	**-0.37***	-0.05	**-0.60***	**0.40***	0.11	**0.66***	**0.36***	-0.02	**0.47***
Caudate	**-0.48***	-0.29	**-0.61***	**0.48***	0.35	**0.57**	**0.36***	0.05	**0.50***
Cerebellum	**-0.47***	-0.18	**-0.68***	**0.49***	0.28	**0.68****	**0.34***	0.15	0.38
Dorsal anterior cingulate cortex	**-0.48***	-0.19	**-0.69****	**0.48***	0.23	**0.72****	**0.34***	0.06	0.43
Hippocampus	**-0.38***	-0.06	**-0.64***	**0.42***	0.20	**0.66****	**0.28***	-0.004	0.40
Inferior temporal gyrus	**-0.48***	-0.13	**-0.67***	**0.50***	0.28	**0.68****	**0.33***	0.03	0.39
Insula	**-0.45***	-0.16	**-0.64***	**0.49***	0.30	**0.67***	0.30	0.04	0.37
Medulla	**-0.46***	-0.26	**-0.57***	**0.44***	0.27	**0.60***	0.22	-0.064	0.30
Midbrain	**-0.37***	-0.04	**-0.56***	**0.42***	0.2	**0.63***	0.25	-0.02	0.33
Middle temporal gyrus	**-0.42***	0.07	**-0.66***	**0.50***	0.25	**0.70****	**0.35***	0.06	0.41
Nucleus accumbens	**-0.46***	-0.25	**-0.64***	**0.48***	0.30	**0.64***	**0.34***	0.02	**0.53***
Orbitofrontal cortex	**-0.48***	-0.10	**-0.73***	**0.51***	0.28	**0.71****	**0.37***	0.12	0.44
Pars opercularis	**-0.52***	-0.17	**-0.74****	**0.53***	0.32	**0.76****	**0.35***	0.07	0.41
Posterior cingulate cortex	**-0.53****	-0.24	**-0.70****	**0.54****	0.35	**0.73****	**0.43***	0.21	**0.47***
Pons	**-0.40***	-0.10	**-0.67***	**0.45***	0.20	**0.75****	0.24	-0.06	0.37
Putamen	**-0.48***	-0.17	**-0.67***	**0.46***	0.26	**0.62****	**0.32***	-0.005	0.42
Rostral anterior cingulate cortex	**-0.42***	-0.11	**-0.67***	**0.43***	0.19	**0.66****	**0.36***	0.08	**0.49***
Superior frontal gyrus	**-0.50***	-0.13	**-0.73****	**0.52****	0.32	**0.72****	**0.40***	0.11	**0.47***
Superior tem-poral gyrus	**-0.44***	-0.08	**-0.62***	**0.51***	0.29	**0.69***	0.27	-0.0005	0.33
Temporal pole	**-0.39***	-0.08	**-0.60***	**0.44***	0.23	**0.65***	**0.32***	0.08	0.40
Thalamus	**-0.45***	-0.17	**-0.67***	**0.45***	0.25	**0.63***	**0.33***	0.18	0.37

Table 4 Associations between the insulin-stimulated brain glucose uptake (BGU) rates at the ROI level and waist-to-hip ratio (WHR), total body fat percentage and visceral (VAT) and abdominal subcutaneous adipose tissue (SAT) masses in the low-risk (LR) and in the high-risk (HR) group. Data are Pearson´s r values. Significant associations are indicates by bold font. * P < 0.05, **P < 0.001.

	WHR		Body fat %		VAT mass			Abdominal SAT mass		
BGU	LR	HR	LR	HR	LR	HR	All	LR	HR	All
Amygdala	-0.38	**0.54***	0.37	**0.58****	-0.04	**0.55***	**0.37***	0.14	0.42	**0.37***
Caudate	**-0.54***	**0.56***	0.28	**0.49***	-0.13	0.41	0.25	0.14	0.31	0.28
Cerebellum	-0.43	**0.56***	0.28	0.36	-0.03	0.36	0.31	0.11	0.19	0.28
Dorsal anterior cingulate cortex	**-0.47***	**0.51***	0.35	0.43	-0.13	0.42	**0.33***	0.13	0.30	**0.35***
Hippocampus	-0.39	**0.48***	0.34	**0.51***	-0.03	0.45	**0.33***	0.16	0.36	**0.34***
Inferior temporal gyrus	**-0.53***	**0.49***	0.40	**0.48***	-0.22	**0.45***	**0.36***	0.13	0.33	**0.40***
Insula	**-0.49***	0.42	0.38	**0.46***	-0.04	0.41	**0.35***	0.24	0.31	**0.38***
Medulla	-0.39	**0.59****	0.31	0.42	-0.13	**0.46***	**0.32***	0.18	0.23	0.31
Midbrain	-0.34	**0.51***	0.34	0.40	-0.05	0.33	0.31	0.23	0.24	**0.34***
Middle temporal gyrus	-0.44	**0.52***	0.39	**0.53***	-0.14	**0.51***	**0.40***	0.11	0.33	**0.39***
Nucleus accumbens	**-0.50***	**0.50***	0.28	**0.51***	-0.08	**0.47***	0.26	0.18	0.35	0.28
Orbitofrontal cortex	-0.46	**0.52***	0.37	0.45	-0.02	0.42	**0.37***	0.20	0.30	**0.37***
Pars opercularis	-0.46	**0.52***	0.38	0.43	0.01	0.42	**0.39***	0.27	0.25	**0.38***
Posterior cingulate cortex	**-0.51***	**0.51***	0.39	**0.48***	0.02	0.44	**0.39***	0.24	0.31	**0.39***
Pons	-0.35	**0.49***	0.33	0.43	-0.04	0.37	0.30	0.20	0.27	**0.33***
Putamen	**-0.55***	**0.59***	0.32	0.43	-0.10	0.35	0.29	0.16	0.26	**0.32***
Rostral anterior cingulate cortex	-0.34	**0.54***	0.30	0.39	0.06	0.39	0.31	0.26	0.24	0.30
Superior frontal gyrus	**-0.50***	**0.54***	0.36	0.48	-0.09	0.41	**0.34***	0.16	0.29	**0.36***
Superior temporal gyrus	**-0.51***	0.39	0.41	**0.53***	-0.16	**0.48***	**0.38***	0.09	0.37	**0.41***
Temporal pole	-0.36	0.46	0.33	0.41	0.01	0.36	**0.33***	0.16	0.27	**0.33***
Thalamus	-0.44	**0.50***	0.29	0.39	0.09	0.31	0.30	0.22	0.22	0.29

LEGENDS TO SUPPLEMENTARY FIGURES

Supplementary Figure 1

Plasma glucose (**a**) and insulin (**b**) levels during the hyperinsulinemic-euclycemic clamp in the low-risk (LR) and in the high-risk (HR) group. Data are mean ± SEM, *$P < 0.05$.

REFERENCES

1. DeFronzo RA, Tobin JD, Andres R (1979) Glucose clamp technique: a method for quantifying insulin secretion and resistance. Am J Physiol 237(3):E214-23. https://doi.org/10.1152/ajpendo.1979.237.3.E214
2. Hung C-S, Lee J-K, Yang C-Y, et al (2014) Measurement of visceral fat: should we include retroperitoneal fat? PLoS One 9(11):e112355. https://doi.org/10.1371/journal.pone.0112355
3. Misra A (1997) Relationship of anterior and posterior subcutaneous abdominal fat to insulin sensitivity in nondiabetic men. Obes Res 5(2):93–99. https://doi.org/10.1002/j.1550-8528.1997.tb00648.x
4. Martinez-Tellez B, Sanchez-Delgado G, Boon MR, Rensen PCN, Llamas-Elvira JM, Ruiz JR (2020) Distribution of Brown Adipose Tissue Radiodensity in Young Adults: Implications for Cold [18F]FDG-PET/CT Analyses. Mol Imaging Biol 22(2):425–433. https://doi.org/10.1007/s11307-019-01381-y
5. Aubrey J, Esfandiari N, Baracos VE, et al (2014) Measurement of skeletal muscle radiation attenuation and basis of its biological variation. Acta Physiol (Oxf) 210(3):489–497. https://doi.org/10.1111/apha.12224
6. Abate N, Burns D, Peshock RM, Garg A, Grundy SM (1994) Estimation of adipose tissue mass by magnetic resonance imaging: Validation against dissection in human cadavers. J

Lipid Res 35(8):1490–1496. https://doi.org/10.1016/s0022-2275(20)40090-2

7. Segal SS, White TP, Faulkner JA (1986) Architecture, composition, and contractile properties of rat soleus muscle grafts. Am J Physiol 250(3 Pt 1):C474-9. https://doi.org/10.1152/ajpcell.1986.250.3.C474

8. Snyder WS; Cook MJ; Nasset ES; Karhausen LR; Howells GP; Tipton HI (1975) Report of the Task Group on Reference Man. A report prepaired by a task group of committee 2 of the international commission on radiological protection. Pergamon Press

9. Soininen P, Kangas AJ, Würtz P, Suna T, Ala-Korpela M (2015) Quantitative serum nuclear magnetic resonance metabolomics in cardiovascular epidemiology and genetics. Circ Cardiovasc Genet 8(1):192–206. https://doi.org/10.1161/CIRCGENETICS.114.000216
