# Supplementary figures and images for: Obesity risk is associated with brain glucose uptake and insulin resistance
Source: Eur J Endocrinol. 2022 Oct 26;187(6):917–28. doi: 10.1530/EJE-22-0509 (PMC9782452; doi:10.1530/EJE-22-0509)

Supplementary Figure 1

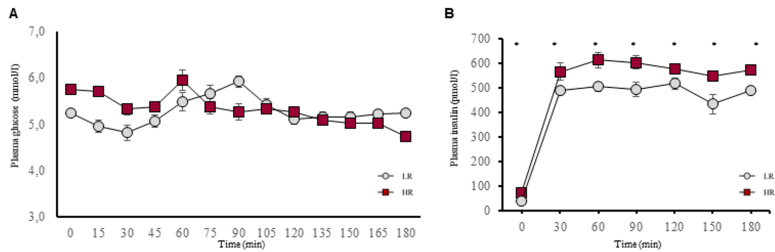

Supplement: Supplementary Figure 1 [file supplementary_figure_1.pdf]
